# The Role of Digital Strategies in Financing Health Care for Universal Health Coverage in Low- and Middle-Income Countries

**DOI:** 10.9745/GHSP-D-18-00271

**Published:** 2018-10-10

**Authors:** Bruno Meessen

**Affiliations:** a Institute of Tropical Medicine, Antwerp, Belgium.

## Abstract

Countries finance health care using a combination of 3 main functions: raising resources for health, pooling resources, and purchasing health services. In this paper, we examine how digital health technologies can be used to enhance these health financing functions in low- and middle-income countries and can thus contribute to progress toward universal health coverage. We illustrate our points by presenting some recent innovations in digital technologies for financing health care, identifying their contributions and their limits. Some examples include a mobile-health wallet application used in Kenya that encourages households to put money aside for future health expenses; an online software platform developed by a startup in Tanzania in partnership with a private insurance provider to give individuals and families the opportunity to choose among different health coverage options; and digital maps by a number of startups that bring together data on health facility locations and capacity, including equipment, staff, and types of services offered. We also sketch an agenda for future research and action for digital strategies for health financing. The development and adoption of effective solutions that align well with the universal health coverage agenda will require strong partnerships between stakeholders and enough proactive stewardship by authorities.

## Abstract (precis)

The development and adoption of effective digital health financing solutions that fit well in both coherent digital health information architectures and the universal health coverage agenda will require strong partnerships between entrepreneurs, developers, implementers, policy makers, and funders.

## INTRODUCTION

There is a strong movement promoting digital technologies as a means of strengthening health systems, in low- and middle-income countries (LMICs), including innovations for demand generation, better management of information, and improved efficiency of health workers.^1^ However, the role and potential of digital technologies in financing health care in the context of health systems strengthening is poorly understood.

To understand the possible role of digital technologies for health financing in LMICs, one must first describe their health systems and identify the health financing challenges they face. In many LMICs, traditionally, the government was responsible for all key health system functions, including priority setting, financing, allocation of inputs, and provision of health services. This is exemplified by the original 1948 tax-funded British National Health Service model in which health services were provided free of cost at the point of care by public civil servants in government-owned facilities. Since the late 1990s, the health sector in LMICs has seen transformative changes consistent with broader societal changes in favor of liberalism, privatization, and citizen emancipation. For instance, in many countries, public-sector facilities have been granted more autonomy and user fees have been introduced. At the same time, the health care market has also seen a growing role of the private sector in service delivery.^2^ Despite these changes, in most LMICs, the public sector still remains the backbone of health service delivery, especially for preventive health services and services that address global health priorities, such as childhood illnesses, HIV/AIDS, and tuberculosis. The private sector, which primarily focuses on curative care, varies in size across countries. For many people, particularly those who are poor and live in rural areas, health care may be provided by a myriad of informal providers delivering services of varied quality.^3^^–^^5^ In nearly all LMICs, ministries of health have been slow to build stewardship capacity to manage this new mixed landscape.^6^ Combined with low levels of government health spending, this can result in a general pattern of health financing where households have to cover a substantial share of the costs. Indeed, in most low-income countries (LICs), only a minority of households benefit from an extra formal financial protection program, such as complementary health insurance. In most middle-income countries (MICs), the proportion of households enrolled in such a financial protection program—usually through employment-based insurance arrangements—is higher but large segments of the population, especially in the informal sector, often remain inadequately covered.

A considerable amount of literature has shown that financial accessibility to health services is a major problem in many LMICs.^7^ While financial accessibility to health services often applies to entire populations, it is particularly relevant for the poorest and the most vulnerable living in these countries. Many poor households decide to curb their use of health facilities or even forego treatment, while others face the risk of impoverishment through so-called catastrophic health care expenditures—out-of-pocket payments for health services that exceed a given fraction of total household expenditure.^8^ Over the last 2 decades, many LMICs, often in partnership with external partners, have tried to address these challenges. A key axis of intervention has been the introduction of innovative health financing schemes, such as community-based health insurance,^9^ social health insurance,^10^ health equity funds,^11^ voucher schemes,^12^ performance-based contracting,^13^ and performance-based financing (PBF).^14^ Many LICs have also opted to remove user fees in public facilities,^15^ often with some targeting for specific vulnerable groups, such as children under the age of 5 or pregnant women,^16^ or high-priority disease areas such as HIV/AIDS or tuberculosis.

With the global spotlight on achieving universal health coverage (UHC) under the Sustainable Development Goals (SDGs), it is critical to identify and scale up effective strategies for promoting equitable access to health services by reducing financial hardships for individuals.^17^ The main purpose of this paper is to explore how digital solutions can contribute to the ambitious agenda of progress toward UHC in LMICs. We have organized the paper around the health financing framework describing the key functions of mobilizing and pooling resources and purchasing services.^18^

## METHODS

To our knowledge, this is the first paper that attempts to discuss digital innovations for health financing in LMICs. As there is not much literature to review, the development of this paper relied largely on our own professional engagement in health financing in LMICs and, to a lesser extent, our own exposure to the digitalization of this health area (see also conflict of interest).

To illustrate possible directions of focus, we provide references to not only existing digital health solutions and tools but also financing schemes that heavily rely on the latest technological developments. They were identified through our own practice, as well as a flash consultation organized on the platform Collectivity (https://www.thecollectivity.org/en/flash_consultations/7).

Clearly, this review is not meant to be exhaustive; rather, the goal of this paper is to map emerging digital health strategies for health financing, identify issues deserving the attention of both the digital and health financing communities, and present recommendations for future action for researchers, digital innovators, governments, and their partners.

## HOW DIGITALIZATION ENHANCES THE 3 FUNCTIONS OF HEALTH FINANCING

Health financing is traditionally conceived of as having 3 key functions: collecting funds, pooling funds, and purchasing health services.^18^ The collection of funds refers to the mobilization of resources to finance health services, with contributions coming from tax payers, employees, employers, public and private donors, and users. The World Health Organization (WHO) recommends that health systems have mandatory funding sources, such as taxes or social security contributions. An important indicator of progress toward UHC is the level of direct, or out-of-pocket, payments for health care.^17^ Payment at the point of care has several drawbacks: it requires cash; it includes a level of uncertainty about the amount to be paid, which deters households from seeking care, especially under fee-for-service systems; and, more fundamentally, it leaves households to cope with the costs without any financial protection.

Health financing is traditionally conceived of as having 3 key functions: collecting funds, pooling funds, and purchasing health services.

The pooling of funds refers to the “accumulation of prepaid health care revenues on behalf of a population.”^18^ To achieve UHC, pooling arrangements need to be established so there are large sustainable pools that allow for the adequate redistribution of financial risk between the healthy and sick and the rich and poor. An important step to address this issue includes reducing pooling fragmentation—the multiplicity of financial pools covering fragmented groups—and duplication, where some people are covered for the same needs by multiple schemes.

Purchasing is the transfer of pooled resources to service providers on behalf of the population for whom the funds were pooled. In many LMICs, health services, particularly those in the public sector, continue to be purchased using line-item budgets. While this method of purchasing is good for containing costs, it represents a passive approach to purchasing health services because it is not related to health or performance outcomes. WHO recommends purchasing to be strategic and to aim for an efficient and equitable allocation of resources to producers of good health.^19^ Next, we describe the current state of evidence for the use of digital strategies for each of these 3 health financing functions.

### Collection of Funds

As explained in our introduction, in many LMICs, a large part of health financing comes directly from the pockets of the users. This is managed through cash payments and paper administration at the facility level. The key digital technology that could transform the collection of funds function is the electronic payment. However, from a UHC perspective, electronic payments will only lead to significant progress if they allow movement from payment at the point of care to prepayment—contribution by the household before the occurrence of the episode of illness—and pooling.

Collecting funds from people who are active in the formal sector does not require major technological developments, as the financial transfer can be organized directly from the employer. Collecting funds from people active in the informal economy is more challenging, as their locations may be unknown, and their administrative existence limited. For this group, the pervasiveness of mobile phones in LMICs and the success of mobile-money initiatives could open up new avenues, especially in settings where the bank system is underdeveloped, costly, or absent.

Collecting funds from people active in the informal economy is challenging, as their location may be unknown and their administrative existence limited.

Kenya is one of the countries currently using mobile money. A study has shown how the mere introduction of the M-Pesa mobile phone-based money transfer, financing, and microfinancing service has already helped households to tap remittances and respond better to health shocks.^20^ M-TIBA (http://m-tiba.co.ke/) is a mobile-health wallet application that builds on this potential by encouraging households to put money aside for future health expenses or to collect contributions from other relatives, such as contributions from adult children to finance elderly parents' health care. It also allows organizations—for instance, a program or a charity—to sponsor targeted households. As providers are enrolled after selection by M-TIBA, it also integrates a purchasing function. By June 2017, nearly 900,000 users were reported registered, giving them access to 450 health facilities, and the system had processed payments totaling $1.4 million for 100,000 visits.^21^

From a UHC perspective, this example highlights at least 2 benefits of using digital technologies for the collection of funds: (1) the possibility to raise funds, outside of formal banking systems and without cash handling, through mobile money (see also experiences in Mali and Kenya)^22^ and (2) the possibility of raising funds from various sources, even across borders—relatives in the diaspora, for instance—for individual or collective use.

It is currently unclear, however, how this potential can be converted into sustainable financing schemes that support UHC. One concern is that if the initiative comes from private investors the focus will be on profitability, which will likely mean that the poorest segments of the population will be overlooked.

The collection of funds has 2 sides: households contribute to a scheme and, in exchange, they receive an entitlement. Some implementation challenges related to the collection of funds and entitlement are: avoidance of fraud (claim for support or reimbursement from people who are actually not covered by the arrangement, with or without collusion between the subscribers and providers); timely updates of the composition of households (birth and death); portability of the entitlement across geographical areas (particularly important for migrant workers); and renewal of insurance policies. Digital strategies may be valuable for overcoming some of these implementation challenges. For example, government-sponsored health insurance programs, like the Aarogyasri Scheme (http://www.aarogyasri.telangana.gov.in/aarogyasri-scheme) in India, use digital technology to maintain electronic health records and prevent fraud by health care providers and patients. The management of entitlements is an area where the gains from digitalization are obvious, as evidenced by systems already in place in high-income countries.

### Pooling of Funds

From a UHC perspective, a digital health solution like M-TIBA has some limitations, as M-TIBA was developed as a medical savings account, not as insurance. The application reminds households to be well-prepared. While it can help family members to handle, and ration, pressing demands from relatives, it does not organize the pooling of funds among subscribers and, therefore, the expenses paid cannot be more than the money uploaded on the account.

A key principle of health financing is that pooling risks between individuals is efficient. As most health shocks are idiosyncratic, meaning they are uncorrelated between individuals, the law of large numbers suggests that setting up a sufficiently large common pool will allow for the removal of uncertainty. A proposition can be made to individuals to pay a certain price, the premium, in exchange for an insurance—the assurance of being protected from the financial risk. Since people are generally risk-adverse, they usually agree to pay the premium.

As most health shocks are idiosyncratic, the law of large numbers suggests that setting up a sufficiently large common pool will allow for the removal of uncertainty.

There are various ways of setting up such pools. Subscription can be compulsory or voluntary. Governance can be under the responsibility of the government, a multi-stakeholder body, private investors, or representatives of the subscribers. About 15 years ago, several nongovernmental organizations (NGOs), bilateral aid agencies, and multilateral agencies explored the feasibility of community-based health insurance (or *mutuelle* in French) and micro-health insurance.^23^ Under such arrangements, households or individuals were invited by an NGO, a social entrepreneurship, or a community group to pay a subscription fee to an insurance scheme, which would then cofinance their health care costs for a given period. Rwanda made huge progress toward UHC with this model,^24^ partly thanks to strong presidential leadership, but replicating this success elsewhere has proven difficult.

The primary problem with community-based health insurance and microinsurance schemes is the high transaction cost to enroll households and collect their premiums, especially in low population density areas, and the selection and monitoring of health providers. Today, some actors take advantage of the pervasiveness of mobile technology and test variations of the voluntary insurance model. Digital tools can contribute in several ways to setting up and managing pools: the Internet can significantly increase access to new subscribers by reducing search costs on both sides of the transaction; as seen in the previous section, smartphone applications can reduce transaction costs for premium collection; and database systems are crucial for managing and processing subscriber data and expense claims from patients and health facilities.

Several private actors are currently developing online solutions that offer insurance packages to households. An example is the software platform developed by Jamii, a startup in Tanzania that partnered with a private insurance provider (http://www.jamiiafrica.com/) to give individuals or families the opportunity to choose among different health coverage options. Interestingly, M-TIBA is aware of the limits of the savings account model, and seems to be heading in this direction as well by setting up a partnership with the National Hospital Insurance Fund of Kenya. Another interesting experience is “My Tonic.com” developed by Telenor Health in partnership with Grameenphone. Through a phone application, the population of Bangladesh can access a wide range of services, including health information, advice, preselection of providers, and health insurance. The most remarkable feature is how the different services are provided in packages designed to empower households to manage their own health, including nudging them toward healthy choices. About 4.5 million people are currently insured through this partnership.

While voluntary health insurance certainly offers a valuable service to their subscribers, from the UHC perspective, it also has a drawback, as it ignores those who cannot afford the premium. Recently, WHO de-emphasized voluntary insurance as a sustainable approach to achieving UHC.^25^ In order to guarantee that everyone is covered, WHO instead recommended countries move toward a predominant reliance on compulsory insurance. However, progress in rolling out compulsory schemes has been variable in LMICs, as it is easier to organize pools within the formal sector than in the informal one. If the market opportunities are real, more initiatives like Jamii or My Tonic will emerge; the trend seems to be particularly strong in the telecom industry. This dynamism should be appreciated not only for the value it brings to households, but also because it invites public authorities and their international partners to move faster on issues, such as stewardship of the sector, and to consider greater use of public–private partnerships.

A particular responsibility for governments is to provide coverage to the poor and vulnerable who cannot contribute. Many countries have set up health financing schemes tailored to overcome specific informational, geographical, financial, and cultural barriers encountered by these groups.^26^ Similar to contributory schemes, a key issue is ensuring the identification of beneficiaries. This is particularly challenging, given that 2.4 billion people in the world have no official identity.^27^ This is an area where the use of digital technologies has been evolving fast. Operators have moved from paper-based registers to digital databases that include pictures and geographic information system (GIS) coordinates of households, and, more recently, to datasets incorporating unfalsifiable identifiers, such as iris scans or fingerprints, for age groups for whom these identifiers work. In this respect, some technological developments look promising.^28^ We anticipate that this will generate new societal questions for the many LMICs that have no advanced experience managing personal data and no established legislation. Interestingly, blockchain technology might open new territories, as it could provide individuals with an immutable identity^29^ independent from state authorities (see the solution created by idbox, http://www.idbox.io/). Yet we anticipate that many governments would want to keep control of the digital identification of entitled households. To that end, a country case to follow is that of India, for at least 3 reasons: it is a democracy concerned with civic and privacy rights, it is embarking on a massive free health care program for 500 million people, and authorities have decided to build this new entitlement program on Aadhaar, a 12-digit unique identity number already used by 1.22 billion residents.^30^

Many countries have set up health financing schemes tailored to overcome specific informational, geographical, financial, and cultural barriers encountered by the poor and vulnerable.

At the household level, the entitlement document may be digitalized from paper to an electronic identity card with chips or scan codes, or even fully dematerialized e-vouchers that are sent directly to the mobile phones of beneficiaries^26^ (see the various solutions developed by RedRose, https://www.redrosecps.com^31^).

The creation of too many siloed health financing schemes can create fragmentation, which is a source of both inefficiency and inequity.^32^ To address that, WHO recommends reducing the number of concurrent schemes in order to reduce fragmentation of offerings for pooling financial resources. Merging pools by itself is difficult and raises several political and technical issues. For instance, merging voluntary health insurance, mainly subscribed to by those with more wealth, with a health equity fund, funded by the government and its aid partners for the health care of the poor, may be challenging because the public resources allocated for the poor might actually finance the use of health services by the wealthy, as the latter have less barriers to overcome.^33^

The creation of too many siloed health financing schemes can create fragmentation, which is a source of both inefficiency and inequity.

In many LICs, insufficient pooling even prevails at the level of public funds; this is particularly true with off-budget external aid. Many health authorities are not aware which geographic areas, health needs, or population groups are covered by several projects and which are underserved. In Uganda, the United Nations Pulse Lab is developing a digital solution for visualizing off-budget contributions from aid actors and identifying funding gaps and overlaps. This highlights the need for the development of computational tools to prepare, implement, and monitor instances of pooling where disparities may be heightened.

In each country, greater convergence and interoperability between data systems used by different schemes will be key. In the absence of prudent stewardship, incompatible proprietary software solutions may become an additional obstacle to the consolidation of pooling. Governments and aid agencies, including WHO, have begun to move toward more unified pools by working on common architectures; agreeing on terminologies and classifications; adopting common identification numbers for users, providers, and health facilities; and requesting interoperability of data systems.

### Purchasing of Services

From a technical perspective, purchasing is probably the most complex and sophisticated of the 3 functions of health financing. Several exciting digital developments can be linked to the move away from line-item budgeting and toward strategic purchasing. The latter is described as a progression on 4 + 1 functions: identification of best value, selection of the right providers, design of smart contracts, efficient enforcement of contracts, and finally, learning, which is the overlying function supporting the 4 others (Figure).^34^

**FIGURE.**
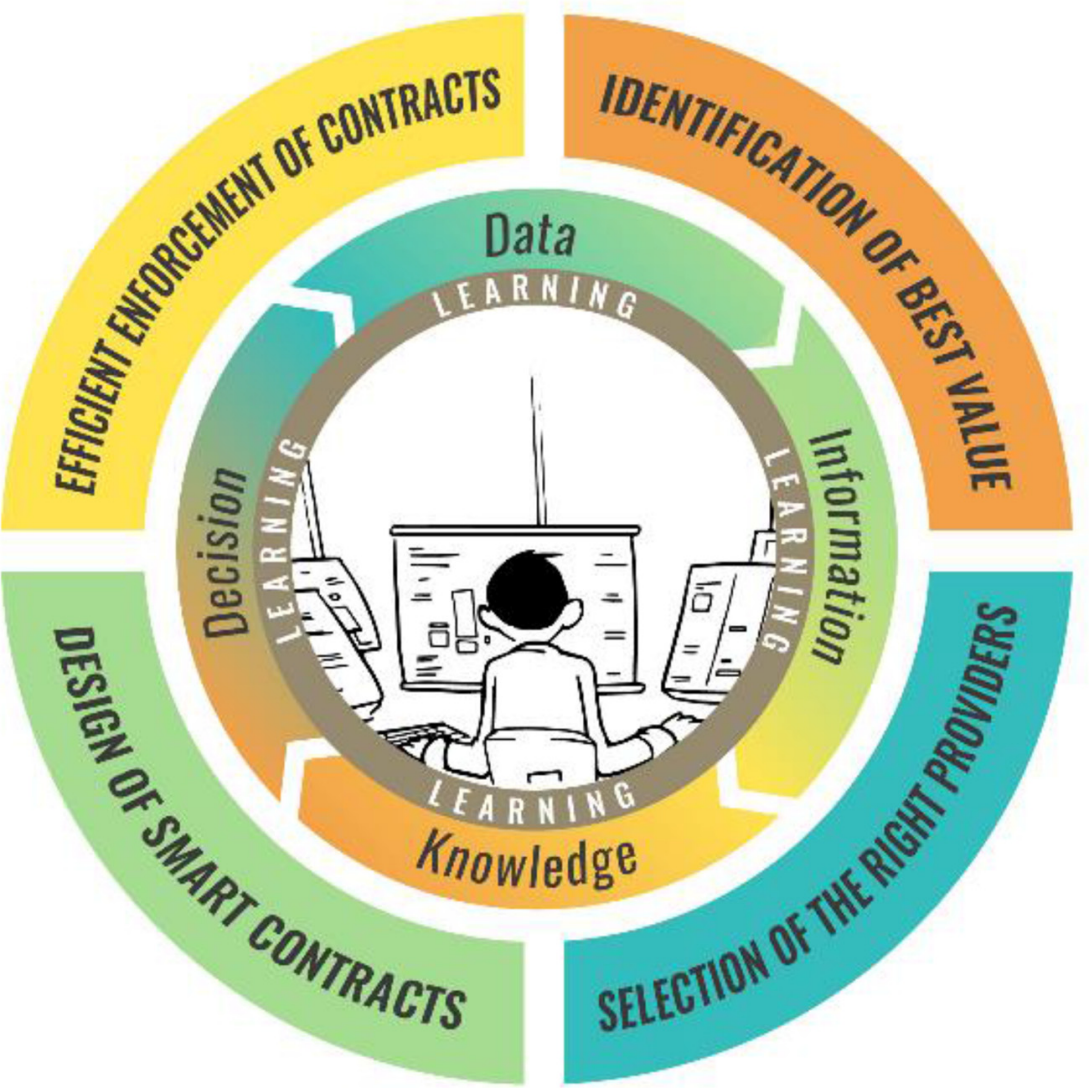
4+1 Functions of Strategic Purchasing

#### Identification of the Best Value

A purchaser has to develop a good and up-to-date knowledge of (1) the population it covers (the number of people) and their health needs (the ‘burden of diseases’), health expenditures, and values and preferences; as well as (2) the interventions and solutions to respond to these needs and demands. This information is vital for determining the content of the benefits package—the set of health goods and services and the conditions to access them—to be provided to the beneficiary group. The main technique to determine the benefit package is cost-effectiveness analysis, a data-hungry evaluation method that uses modeling and results from clinical trials. Whereas this field of global health is dynamic in terms of methodological developments,^35^ digital innovations seem to have been occurring at a slower pace.

Today, as far as LMICs are concerned, the most visible digital players in these areas are global actors. However, health financing is not their primary objective; their main aim is to establish burden of diseases (see http://www.healthdata.org/) or gather aggregate data on health services (on primary health care, see https://phcperformanceinitiative.org/). Their use of digital technologies is focused on compiling and processing data, producing forecasts, visualizing analyses, and sharing these products with stakeholders, often with a limited view of the actual needs of national strategic purchasers. A pioneering step toward linking data with the actual allocation of resources by ministries of health in LMICs was the Microsoft Excel-based marginal budgeting for bottlenecks tool jointly developed 15 years ago by UNICEF and the World Bank.^36^ The more recent alternative, developed by WHO, is the OneHealth tool (http://www.who.int/choice/onehealthtool/en/), which not only links strategic objectives and targets of disease control and prevention programs to the required financial investments but also includes scenario analysis.

WHO's OneHealth tool links strategic objectives and targets of disease control and prevention programs to the required financial investments.

We expect further developments in this area in LMICs. One driver will be the growing number of LMICs developing their own health technology assessment capacity. Another driver will be the progressive use of so-called real-world data—data obtained not from randomized trials but from more heterogeneous groups of patients already under treatment or observation.^37^ In the future, strategic purchasers will make greater use of granular data on population and health services. The main challenge will probably remain the same as described earlier: solutions will have to combine data of very different natures from very different sources. This will require the development of governance models that secure participation from the different public and private stakeholders needed to create real value for purchasing bodies and the whole health system.

#### Selection of the Right Providers

With the growing pluralism of the health sector in LMICs, the choice of possible providers is also growing. As mentioned in the introduction, in many LMICs, private for-profit facilities are loosely regulated and often not included in the national health information system. In this context, it will be up to the purchasers to identify, screen, select, and enlist the facilities that will deliver the benefit packages covered by the financing scheme. Many elements of information can help purchasers to make informed choices. Yet collecting and analyzing such data entails huge search costs; this is typically an area where digital solutions can bring major gains. First, purchasers need to locate private health facilities and determine what services they provide. Startups such as Healthsites (https://healthsites.io/), Blue Square (https://bluesquarehub.com/), and World Pop (http://www.worldpop.org.uk/) are developing digital maps that will bring together data on locations, health facility capacity (equipment, staff, package of services), and, soon, households (number, needs, socioeconomic status, and so on) in the catchment areas. They tap different opportunities, such as crowdsourcing, GIS, high-resolution satellite imagery, and, perhaps soon, big data from mobile network operators.

Second, purchasers also have to think system-wise and be ready to consider ‘provision’ beyond health facilities only. For some needs, this requires opting for a whole chain of service providers, including sometimes the users themselves. For instance, Vodaphone developed an Uber-like mobile phone application to organize payment of taxis for transporting women to health facilities for delivery in Tanzania.^38^ Another example of such an approach is Tiko, a social marketing solution that tries to incentivize the uptake of healthy behaviors (http://triggerise.org/). Third, for any service provider, whether a hospital, taxi driver, or community health promotor (see, for instance, https://livinggoods.org/), the capacity to guarantee quality standards in the benefit package is a central component of the purchaser's value proposition to users. There are various ways of collecting information, assessing performance, and recognizing and rewarding technical capacities, such as certification, franchising, and accreditation. One could, for instance, imagine combining information collected from users through a crowdsourcing application—such as the Mera Aspataal application introduced by the Ministry of Health of India, which has already generated more than 2 million user feedback comments about 1,084 hospitals (http://meraaspataal.nhp.gov.in/)—with an evaluation produced by mandated professional evaluators. Collecting continuous information about the performance of providers, analyzing it, and taking action will be major areas of development in the years to come.

#### Design of Smart Contracts

The health sector is primarily a service sector. In economic terms, this means that most of the transactions are of the principal–agent relationship type: one party pays another party for a job to be done under uncertainty.^39^ In this type of relationship, the principal defines and measures as accurately as possible, and at the lowest cost, the service it expects to get. Uncertainty can blur the attribution of the results and an asymmetry of information may allow the agent to shirk, underproduce, or save on costly attributes of the service under purchase. Over the last decades, technology—such as tablets, digital cameras, and cloud computing—has enabled a substantial reduction in the cost of collecting and reporting information on human behaviors and resources, including services delivered by health providers. This has encouraged the emergence of new provider payment methods and their expansion worldwide. For instance, today, more than 30 LMICs are piloting or scaling up PBF schemes. PBF compensates health facilities according to the quantity and quality of services they have provided to the population.^40^ Digital technologies allow the implementation of contracts with sophisticated definitions of performance and tailor them to the behaviors of targeted providers and the users themselves. New data collection techniques—such as tablets, Internet of Things (physical devices, such as refrigerators for vaccines, that are embedded with electronics, software, and sensors, allowing them to connect and exchange data), and wearable health technology—are expected to increase the granularity of collected data and improve contractual terms in the future.

Uncertainty can blur the attribution of results and an asymmetry of information may allow agents to shirk, underproduce, or save on costly attributes of the service under purchase.

#### Efficient Enforcement of Contracts

The execution and payment of contracts requires checking whether the providers deliver; monitoring how they adapt to incentives, which includes detecting fraud; and processing payments. The cloud-based solutions developed for PBF illustrate well how technology is a key enabler of contract enforcement. For example, Open Results-Based Financing (OpenRBF) software (http://www.openrbf.org/) allows PBF purchasing agencies to enroll facilities, define performance indicators, collect performance data from facilities, verify the data, organize payments to facilities, produce invoices for the scheme sponsors, produce an analytical dashboard, and report publicly on health facility performance. Efficient enforcement of contracts also requires leveraging synergies with existing data systems. A new version of OpenRBF has been developed on the District Health Information System 2 platform (https://www.dhis2.org/), which is used in many LMICs as a routine health information management system. Another way to be efficient is to reduce redundancies in paperwork. Under output-based payments, verification of data and activities is necessary, as performance-based contracts can result in providers overreporting activities or inventing fake users. A growing number of PBF programs use tablets for data verification: field verifiers enter data during their visits of health facilities and households and then transfer the data to the purchasing agency. Verification in PBF programs can be costly; one proposal is to move from exhaustive verification to risk-based verification.^41^ Benchmarking and algorithms are expected to help to identify facilities presenting a risk, such as outlying performance. More fundamentally, digital progress elsewhere in the health system—unique identifiers for each user, better electronic patient records, and direct access to patients through phones—will significantly reduce the need for physical verification.

Traditionally, principal–agent models assume that principals are loyal contractors who respect contractual terms. In LMICs, this is a strong hypothesis: the purchaser—governments, in particular—can encounter budgetary or cash-flow problems leading to delayed payments; for example, its own health or treasury administration can also be unreliable and inefficient. Digital tools can limit opportunism by the principal by enhancing the integration of the payment system with the banking system, moving to mobile payment^22^^,^^42^ and maybe, in the future, using blockchain solutions to log transactions, such as the one developed by the startup Disberse (http://disberse.com). At the same time, open digital dashboards, such as Data Viz (https://bluesquarehub.com/services/data-viz/), can enhance transparency and—in limited circumstances, such as the presence of trained data journalists or activists—accountability.

#### Learning

Learning is a crosscutting function at the heart of purchasing and of UHC piloting, more broadly.^43^ In many LMICs, paper-based systems introduced by different schemes or vertical programs have led to the fragmentation of data and underutilization of information for governance and resource allocation. In reality, data-driven learning requires openness, proactivity, curiosity, analytical skills, time, and readiness to change our own and others' preset notions at the risk of engaging in disagreements and conflicts. Digital strategies can help automate the learning process, especially when iterative data analyses are involved. Several examples exist in the digital health world where program managers and various stakeholders can view real-time data via smart dashboards and data visualization software. Algorithms to aid decision making—such as a recommendation to change the price for some services to boost their production or identifying and handling outlier facilities—and artificial intelligence will feature more as technology evolves. We highlight the importance of developing collective intelligence within the international community of health financing experts. Digital tools have facilitated the setup of communities of practice and expert networks, such as the Harmonization for Health in Africa Communities of Practice (http://www.healthfinancingafrica.org/) and the Joint Learning Network (http://www.jointlearningnetwork.org/).^44^ The PBF Community of Practice, which currently has more than 2,000 users, is, for example, the leading user of the collaborative platform Collectivity (http://www.thecollectivity.org/).

Digital strategies can help automate the learning process, especially when iterative data analyses are involved.

## DISCUSSION AND CONCLUSION

Institutional arrangements are key to not only structuring transactions but also to achieving the overall collective action required for progress toward UHC. The main objective of this paper was to explore how digital solutions could contribute to the effectiveness of health financing institutional arrangements in the context of LMICs and to recommend future directions for research and innovation in this space. We illustrated health financing issues and solutions using digital health strategies that are already in place or are currently being piloted, in order to highlight some key principles for the development of a fair and efficient health financing system. In the process, we presented our critical appraisals of some recent digital health financing strategies and highlighted gaps that need to be addressed, especially with respect to equity concerns, in order to consolidate the UHC agenda. Obviously, achieving UHC is a long-term endeavor, requiring action at both the global and country level. The main challenges to reaching UHC are not technological, they are political, financial, and organizational.^43^ While the contribution of digital solutions is partial and dependent on progress made by the other building blocks of the health system, digital solutions can still play an important role as enablers and levers for change.

While the contribution of digital solutions is partial and dependent on progress made by the other building blocks of the health system, digital solutions can still play an important role as enablers and levers for change.

The Box provides a list of possible gains generated by digital solutions for health financing. This list should be interpreted with caution. In this review, we have not provided scientific evidence to support the effectiveness of the digital solutions under consideration; to our knowledge, such evidence does not exist. This is obviously a major limitation of our work, and it inspires 3 main reflections.

BOX **Untapped Benefits of Digital Solutions for Health Financing**

At several levels, digital solutions can contribute to health financing arrangements that support moving toward universal health coverage. They can:

- help users and their relatives overcome cash constraints and the underdevelopment of the bank system (with mobile-money)
- reduce many transaction costs, such as identification of beneficiaries and fast processing of payments, when parties have to contract, such as households with insurers and purchasers with providers
- save time and reduce administrative costs by more efficiently capturing and transferring data by removing, for example, hard copies
- enhance computation of economic data, allowing for more accurate estimates and advanced analysis
- allow a larger pool of providers by structuring the screening of their capacities and creating economic incentives for them to join the pool, such as giving them access to more patients
- allow new contractual terms by capturing and transferring more granular information about users and providers and thus reducing asymmetry of information at different levels
- consolidate trust between parties by allowing greater transparency and accountability
- allow new kinds of analysis, such as benchmarking
- enhance decision making with models and algorithms making use of big data
- allow new kinds of collective learning processes through online communities of practice
- allow the emergence of new actors—such as entrepreneurs and startups—and service providers, bringing more competition and innovation to the overall health financing sector

First, we posit that this lack of scientific evidence does not equate to a lack of knowledge, learning, and evidence. Those who work in the digital industry do focus on learning and validating their innovations in digital strategies for health financing. Investors back innovators to the extent that their solutions gain enough ‘traction’ from customers, including revenue generation. In search of market validation, entrepreneurs implement fast learning cycles^45^: they are not afraid to fail, as long as they fail fast and can learn, adapt, and ‘pivot.’ As much as possible in our review, we provided figures about customers adopting applications and countries using innovations for their digital strategies; for digital entrepreneurs, these are the key metrics to focus on. Willingness to pay is a strong indication of value creation.

Second, more scientific research on digital strategies for health financing is needed. Enthusiasm and ‘disruptive’ vision are important drivers for social change, but there are at least 2 reasons to advocate for more critical assessment by scientists: (1) some digital solutions are promoted by their developers as generating benefits for vulnerable groups with limited collective agency and (2) most solutions aim at public budgets for their long-term funding. It is crucial that long-term health impacts are proven; however, an appropriate research agenda has yet to be developed. Some of the gains digital solutions bring are obvious—for example, digital platforms improve the quality and timeliness of data—and do not seem to require research. For other aspects, it is sometimes difficult to isolate the actual contribution of the digital solution from the health financing scheme in which it is embedded. For instance, countries will rarely consider implementing a PBF scheme without a data system to process the claims by the health facilities. In such situations, the cost-effectiveness of the digital tool is closely linked to the PBF scheme itself. But some other gains deserve special scrutiny in the context of UHC. For instance, does the digitalization of a voucher scheme affect the equity of the program; meaning, does it help the most vulnerable populations gain access to the scheme? Does the adoption of tablets for data collection at facility level or biometric-based recognition for user authentication reduce the cost of verification in PBF programs?^46^

Third, we recommend establishing a more proactive stewardship of the overall learning and action agenda on digital solutions for health financing. Several questions will not be resolved by empirical research, but rather through systemic analysis, coherent vision, and coordinated action. We believe that key international organizations like WHO can take a stronger stance on issues such as governance models, interoperability and integration (also, with solutions serving other building blocks of the health system), open source solutions, and open data. Aid agencies have an important role to play beyond funding new technology developments and pilot studies at the country level. They can promote principles and good practices (see, for example, the 10 principles of donor alignment for digital health at http://digitalinvestmentprinciples.org/) as they have the resources and the prescriptive authority to encourage the development of open source solutions, versus the purchase of proprietary systems. A good example of that is the open source health insurance management information system (openHIMIS) developed by the Swiss Tropical and Public Health Institute for the management of district-based community health funds in Tanzania, and is now an open source solution that is available to other countries.^47^^,^^48^ Aid agencies could also support the emergence of a stronger and more cohesive digital health ecosystem.

The development and adoption of effective solutions that fit well in coherent digital health information architectures will require strong partnerships between entrepreneurs, developers, implementers, policy makers, and funders. One issue will be to find the right balance between creating opportunities for entrepreneurs from the digital industry and protecting LMICs from being trapped in expensive and out-of-control digital developments.

LMICs have to develop their own vision for digitalizing the financing of their health sector; however, they will need to pay particular attention to technological path dependencies, as data systems might be a constraint when it is time to merge risk pools.

For all these actors, there are interesting momentums to seize: several SDG targets, including those outside the health SDG, such as SDG target 16.9 on providing legal identity for all, could structure our global efforts. We hope that this paper will convince some actors to join forces for better solutions for health financing.

## Notes

**Peer Reviewed**

**Funding:** This work was made possible by a grant from the Aetna Foundation, with support from the World Health Organization and The Johns Hopkins University Global mHealth Initiative.
